# Importance of therapeutic patient education in ichthyosis: results of a prospective single reference center study

**DOI:** 10.1186/1750-1172-8-113

**Published:** 2013-08-01

**Authors:** Helene Dufresne, Smail Hadj-Rabia, Charles Taïeb, Christine Bodemer

**Affiliations:** 1 Department of Dermatology, Reference center for genodermatoses and rare skin diseases (MAGEC), INSERM U781, Université Paris Descartes - Sorbonne Paris Cité, Institut Imagine, Hôpital Universitaire Necker-Enfants Malades, Paris, France; 2 Public Health and Quality of Life, Pierre Fabre, Paris, France

**Keywords:** Genodermatoses, Therapeutic patient education, Children, Adults, Ichthyosis, Collective program

## Abstract

**Background:**

Ichthyoses are a heterogeneous group of rare genodermatoses. Patients and their families face difficulties related to daily care and management that may be aggravated by social isolation.

**Objectives:**

To evaluate the impact of therapeutic educational programs in improving the knowledge of ichthyosis patients, and their relatives, about their disease.

**Patients and methods:**

We organized a two sessions-program of “know-how” dedicated to the overall management of ichthyoses. These sessions were conducted based on a tool specifically designed for the study, which addressed our various areas of expertise through a collective game. The participants (patients and their parents and siblings) were divided into groups, and the questions were tailored according to the participants’ age. The program was conceived as a knowledge reinforcement program that took place during a weekend of education and rest, organized away from healthcare structures. Our aim was to facilitate the program in a neutral place to encourage respite care and to ensure the availability of a multidisciplinary healthcare team.

**Results:**

After the reinforcement session, children aged from 6 to 12 years and their families acquired the targeted know-how and social skills.

**Conclusion:**

Benefits of TPE in the management of ichthyoses are the following: (1) the trust between patients their families and the caregivers was strengthened; (2) the context of the program encouraged self-expression, answered questions and provided mutual aid; and (3) the more self-sufficient families could better manage emergencies.

## Background

Inherited ichthyoses form part of a large, clinically heterogeneous group of Mendelian Disorders of Cornification, which usually involves all or most of the integument. They are characterized by chronic -mild or severe- dryness, hyperkeratosis and scaling. Individually, they are rare and considered as orphan diseases. Ichthyoses seriously impact motor development during childhood. In particular, restricted movements, as well as sensory impairment (ectropion and ear plugs) are common [1]. The essential care, often restrictive, is a daily burden, but it allows children to grow up, in the most appropriate conditions. Nowadays, therapeutic patient education (TPE) is considered as an essential tool in the management of ichthyoses.

The use of TPE varies depending on the healthcare system, area healthcare provisions, and on the mindsets of healthcare professionals and patients [2-4]. In France, a law reforming hospitals included TPE in the course of care [5] and the core value of therapeutic educational programs is now being recognized through its recent accreditation by the Regional Agency of Health. Several studies have revealed that daily consultations are limited in providing correct counseling on how to manage the disease for patients [6-8].

In fact, pediatric publications in the field of TPE has been on the raise in the last decade, although they have mainly focused on asthma and diabetes [9-11]. In dermatology, TPE has been mainly used in atopic dermatitis and psoriasis [12]. In our center, the monitoring of a large cohort of children with ichthyosis revealed a strong sense of exclusion among these families, as well as common misunderstandings about the disease [13]. Therefore, we decided to implement a reinforced collective TPE program targeting children with ichthyosis and their families that have previously achieved an initial individual TPE program. Here we report our experience in patients with ichthyosis an their relatives who beneficiate of a specific program consisting of two sessions based on a game with a set of multiple-choice questions. We emphasize a close correlation between social isolation and therapeutic difficulties, as well as the importance of initial and reinforced TPE programs designed according to different age groups, but also for parents and siblings.

## Patients and methods

Children aged above six years with a clinically and/or molecularly confirmed diagnosis of ichthyosis who were regularly assessed in the center and had already achieved the individual initial TPE session were contacted to participate in the weekend of therapeutic education focusing on knowledge reinforcement. Individual initial TPE sessions focused on pathophysiology of chronic disease, importance of daily care and on management of pruritus. The parents and the siblings of each included child were included in the program.

Given the limited capacity of the reception center (Avène® Spa), the families included were the first 31 families who confirmed their interest in the program and fulfilled the inclusion criteria. Written consent for the publication of the study results was taken from patients and their families.

Before the weekend, educational diagnosis was performed by the same interviewer on each of the patients and their families, through a standardized questionnaire designed by the multidisciplinary MAGEC team. Educational diagnosis is the first step of the educational process. It is a systematic comprehensive, iterative collection of information by the health care provider concerning the patient’s bio, clinical, educational, psychological and social status. This information is to serve for the construction of individualized TPE. An educational diagnosis is made individually for each patient and leads to define the goals tailored to each patient. It was used to assess the level of knowledge and autonomy of the respondents and to set goals which were divided into three fields: knowledge, know-how and social skills.

A percentage of less than 50% of correct answers was an inclusion criterion. To facilitate the progress of the program, a neutral place which was away from hospital was chosen. The aim was to encourage respite care and guarantee availability of the multidisciplinary healthcare MAGEC team.

Two sessions of two hours each, called 123 Tem’peau sessions, led by a physician and a paramedic team member, addressed the following issues/questions:

- For affected children and their siblings aged 6 to 12: “What is ichthyosis? Why do I need the cream? Why am I sick? Is it normal to have pain? What about school and me? What about the hospital and me?” (session 1)

- For parents, the affected children and siblings (age > 12): “What is ichthyosis? What are the treatments? What is genetics? What is functional management? What are my social rights?” (session 2)

A game with a set of multiple-choice questions was used. It addressed various topics: therapy, genetics, care, pain, rehabilitation and social rights. This tool is designed for collective TPE performed as a fun team game. The game’s aspects are underscored by hints.

The matrix of the skills assessment of the patients (session 1, Table 1) and their parents (session 2, Table 2) included in the program and the verification of their achievements are shown. Both, skills assessment and educational diagnosis were performed by the same interviewer. The assessment table was completed during interviews with the children and their parents, two months after the respite care weekend.

**Table 1:** Assessment matrix of the skills of children with ichthyosis after a therapeutic education session

***Skills***	**Therapeutic Evaluation 1,2,3 Tem'Peau**	**True**	**False**
***Knows how to communicate about his illness***	I can tell my doctor that I did not understand what he said.		
	I can tell my doctor that I've had enough of the cream.		
***Facilitate his integration in school***	I cannot paint the same way my friends do.		
	If in school, they mock me, I can tell an adult.		
	If my parents and my doctor agree, I have the right to go on a trip organized by the school.		
***Knowledge about his treatment***	I must put some moisturizer on once a day.		
	The cream is used to moisturize my skin.		
	Fucidine®(fusidic acid) is a moisturizing cream.		
	There is nothing to do about the itching.		
***Knowledge about his disease***	Ichthyosis can affect the skin of my whole body.		
	My disease also concerns my eyes and ears.		
	I can't transmit my disease by touching my friends.		
	Ichthyosis is a genetic disease.		
***Knowledge about the analgesic treatments***	EMONO - Kalinox® is an inhalable drug used to reduce pain.		
	It is necessary to wait for very strong pain before taking a painkiller.		
	It's normal to suffer pain because I'm sick.		
	If the analgesic treatment is ineffective, I can increase the dose myself.		

**Table 2:** Assessment matrix of the skills of parents of children with ichthyosis after a therapeutic education session

***Skills***	**Therapeutic Evaluation 1,2,3 Tem'Peau**	**True**	**False**
***Knowledge about social rights***	The Memorandum of Special Reinforcement (100%) is an allowance paid for by the MDPH.		
	My child, who is affected by ichthyosis, can benefit from the Education Allocation of Handicapped Children.		
	There is no leave available to temporarily stop working to take care of the sick child.		
***Knowledge about the measures to facilitate the enrollment***	PAI means individualized support and integration plan at school.		
	It is necessary to build an MDPH case to be granted a special needs assistant.		
	When the child is absent for medical reasons, he is entitled to educational assistance at home.		
***Knowledge about the scope of the various professionals***	The social worker can renew a care order.		
	The rehabilitation exercises help to maintain good mobility.		
	An occupational therapist can assess the speed of writing.		
***Knowledge about pain management***	At the hospital, my child may benefit from EMONO - Kalinox® to avoid pain during his cures.		
	I can increase the dose of analgesics without medical advice.		
***Identifying the various topical treatments***	Emollient creams are needed to moisturize my skin.		
	A single tube of therapeutic cream can be used for the whole family.		
	The moisturizer does not expire.		
	Therapeutic creams and moisturizers are used in the same way.		
***Knowledge about the disease***	Ichthyosis is due to a thickening of the hypodermis.		
	The application of cream can relieve itching.		
	I can ask for an emergency appointment with a physician if I find my child's skin unusual.		
	In one family, there are several possible modes of inheritance.		
	There are ichthyoses for which the mutated gene is not known.		
	Anyone can benefit from genetic counseling.		
	Children with ichthyosis shouldn't be monitored only by hospital doctors.		
	Because of their disease, children affected by ichthyosis can not enjoy playing music.		

## Results

### Population

In total, the number of participants was 82, including the children’s parents and siblings. Thirty-one affected children aged between 6 to 15 years old participated in the study (mean age, 9 years). The group consisted of 14 girls and 17 boys. Clinically, they suffered from Netherton syndrome (seven boys and five girls), keratinopathic ichthyosis (two boys and four girls), lamellar ichthyosis (six boys and four girls), congenital ichthyosiform erythroderma (one boy and one girl), and ichthyosis follicularis, atrichia, and photophobia (IFAP syndrome, one boy). Twenty-one cases were sporadic. The other ten belonged to the same three families. They were accompanied by 44 parents (26 mothers and 18 fathers, mean age, 36 years for both parents, 31 years for mothers and 39 years for fathers), and their unaffected siblings (four girls, three boys, mean age 11 years). None of the parents suffers from ichthyosis.

Knowledge before the session (educational diagnosis). The main data are reported in Tables 3 and 4.

**Table 3:** Reinforcement by therapeutic education of children with ichthyosis

	**Skills**	**Before**	**After**
Knowledge	Know the name of their disease.	30 (96.7%)	31 (100%)
	Know how to explain why they are ill.	25 (80.6%)	31 (100%)*
	Know the name of the treatment.	22 (70.9%)	27 (87.1%)
	Know why they go to see certain specialists.	2 (6.4%)	31 (100%)*
Know-how	Think that there is no treatment against the itching.	5 (16,1%)	3 (9.6%)
	Know what the treatments are for.	9 (29%)	29 (93.5%)*
	Know why they have to apply cream every day.	23 (74.2%)	30 (96.7%)*
	Think that Fucidine® (fusidic acid), an antibiotic cream, is a moisturizer.	3 (9.6%)	1 (3,2%)
Social skills	I can tell my doctor that I've had enough of the cream.	17 (54,8%)	30 (96.7%)*
	Think that “it is not normal” to suffer pain.	12 (38.7%)	7 (22,5%)
	Think that they should not tell the doctor that they didn’t understand what he said.	11 (35.4%)	0 (0%)*
	If someone mocks me, I will tell an adult.	9 (29%)	22 (70.9%)*

Educational diagnosis is in the first column. The answers to the assessment matrix (see Table 1) are in the second column. *Significant. results.

**Table 4:** Reinforcement by therapeutic education of parents of children with ichthyosis

	**Skills**	**Before**	**After**
Knowledge	Know the difference between a moisturizer, an antibiotic cream and local corticoids	33 (75%)	38 (86.3%)*
	Know how to store the creams and their usage expiration	7 (16%)	26 (59.1%)
	Know that ichthyosis is a genetic disease	32 (72.7%)	44 (100%)*
	Know the recurrence risk of the disease	15 (34.1%)	22 (50%)*
Know-how	Know how to recognize dangerous situations	24 (54.5%)	39 (88.6%)*
	Know what to do to manage the itching	11 (25%)	27 (61.3%)*
	Know why their child goes to an occupational therapist	25 (56.8%)	39 (88.6%)*
	Know how to adapt the treatment to the skin’s state	24 (54.5%)	34 (77.2%)*
Social skills	Know that they can benefit from a special allocation for their child	39 (88.6%)	44 (100%)*
	Know the aid options at school	25 (56.8%)	42 (95.4%)*
	Know whom to resort to in case of an emergency	29 (65.9%)	44 (100%)*
	Know how to explain the disease at school	11 (25%)	26 (59.1%)*

Educational diagnosis is in the first column. The answers to the assessment matrix (see Table 2) are in the second column. *Significant. results.

The children had difficulties to explain the origin of their disease, more specifically, the concept of "genetics" and the need for multidisciplinary consultations as part of their medical management (only 6.4% of the children knew why they had seen an ENT specialist and an ophthalmologist). Also, the children had difficulty to explain the measures taken to improve the pruritus (16.1% of children thought that there was no possibility of improving the pruritus). A large percentage of children (35.4%) were afraid to tell the physician that they did not understand what was discussed, 54.8% thought that they should not express lassitude to the treatment and 71% could not explain the purpose of their treatment.

For parents, educational diagnosis revealed difficulties mostly in different area such as: understanding the concept of "genetics" (27.3%), the usage of treatments (only 16% knew how to store the drugs), the management of pruritus, which remains very "disheartening" (25% knew how to develop a strategy to manage the itching), and communication with the school (75% did not know how to explain the disease to the school).

In affected children, the skills acquired after the 123 Tem’peau session (session 1) have a good level of significance in the fields of knowledge, know-how and social skills (Table 3). For the parents, the significant results after the 123 Tem’peau session (session 2) according to the areas of expertise are reported in Table 4.

## Discussion

The experience of therapeutic education in the management of children with ichthyosis seems important to share while publications are limited. The establishment of a TPE group which was constituted by health professionals seems mandatory because of a the strong sense of rejection expressed by the patients and their families, the impact on quality of life associated with the misunderstanding of the disease and the importance of multidisciplinary monitoring [10,13].

In our study, we used a step-by-step approach. In brief, TPE group in close partnership with patients and their families identified the required skills for better daily management of the disease. An interview guide for educational diagnosis was created, and learning objectives specific to ichthyosis were determined. This stage of educational diagnosis was essential for better setting the difficulties and the actual resources of patients and their families before going further with the reinforcement program. The areas of need were noticed immediately among 31 families and consist of difficulties with social relationship, possible functional, motor and sensory impairment, and management of pruritus.

In general, patients with ichthyosis have common physical appearance, with typical facial aspect and hand roughness, sometimes associated with peculiar and unpleasant odor and skin peeling. Ichthyosis patients are thus often regarded unclean, as potentially contagious. This create a feeling of guilt in the affected children, who clearly don't dare ask any questions to the doctor nor call an adult. While in most cases the parents were aware of specific healthcare benefits for their children (88.6% of cases, Table 4), many were unaware of the availability of possible specific help at their schools (43.2%). This results in both the children and the parents in a feeling of inability to explain the disease to a community such as the school (Tables 3 and 4).

Apart from social difficulties, lack of skin flexibility can limit joint movements, prevent fingers to grow, interfere with fine motor skills, walk and grip due to skin cracks. Prevention of retraction or poor posture, is particularly important in children. Understanding how indispensable a multidisciplinary and regular management is crucial for the growing child. Neither the children nor their parents had understood the prevention of these motor and sensory complications.

Educational diagnosis has helped to define the expectations of a TPE reinforcement program and its required tools. The goal of therapeutic education of affected children is to help them to better understand their illness, the necessary treatments for their survival and comfort, and to perform their daily skin care. To achieve our goal, the multidisciplinary team of MAGEC center decided to elaborate a collective instead of an individual TPE program, away from a traditional healthcare setting, and designed as a fun tool. We focused on the educational value of classical learning in children through playing. We also emphasized the importance of help and support from the group, the emulation related to it, and on the positive value of self-esteem for the child, so that it would increase the patients’ "trust capital”. From the very first beginning of this program, it appears that this experience was positive, thanks to the special moments of communication that allowed throughout the weekend. We can also note from our results, that the patients and their parents acquired most of the objectives of this program especially regarding the fields of know-how and social skills (Tables 3 and 4).

However, further reinforcement sessions will be needed, such as the different options for pain management, which did not appear to be acquired at the end of the program. We acknowledge our evaluation tool may be improved. At a first sight, not everyone understood the word “analgesic” when used in the questions and it seems mandatory to find correct simple words for an optimal communication between caregivers and patients. Also, many products were only known to the families under their brand name. Therefore, further sessions will optimize tools regarding these items and patients and their families need to be more involved in the construction of these tools.

The mode of inheritance is also not always clearly understood. Several factors may explain this difficulty. The first is the heterogeneity of the mode of inheritance of ichthyosis in the patients' group. Each family, however, understood how its own syndrome/disease was transmitted. The second is the personal, intimate, or confidential aspect, in which parents often felt guilty of the "heredity" of the disease and thus refrained from asking questions in the framework of a group program. The issue of genetics must be treated in further individual TPE reinforcement sessions.

Seven unaffected siblings (mean age, 11 years old) were associated with this TPE program mostly for reasons of practical family organization. However, the experience confirmed the importance of holding TPE sessions for siblings as well, allowing a better control of a chronic disease that impacts the whole family history. Brothers and sisters ask themselves many questions but receive little information about the illness of their affected sibling. Therefore, specific TPE sessions may be proposed to siblings which should focus on personal need, i.e. genetic aspects of the disorder, and general information useful for social relationship.

In addition, a specific session for teenagers was deemed essential. For logistical reasons, we chose to include the six children, patients and siblings, over age 12 with the parents’ group. Teenagers’ participation was interesting, but we observed a resistance related to the nature of that particular group.

Overall, this original group program was a success for all participants, as shown by the families' responses to the satisfaction questionnaires (data not shown).

Most of the objectives set during the educational diagnosis were achieved throughout the program. This significantly helped the affected child (Table 3), but also his siblings (data not shown), to better understand the disease, better assert and defend himself when he suffers from his illness and the associative mockery. The aim of the treatments was better understood, the vigilance of adults regarding motor and sensory impairment were more motivated and the manipulation of treatments was improved. The dimension provided by the group was instrumental in removing the isolation of families and facilitated the acquisition of effective resources in the field of social integration. Clearly, a TPE program should include initial and reinforcement sessions supported by the analysis of educational diagnostics, and repeated with a frequency depending on the chronicity of the disease. In these diseases of neonatal origin, parents and siblings should be given the same attention as the patients. Also, the period of adolescence cannot be assimilated to that of childhood or adulthood, and requires specific and specially tailored TPE programs.

Finally, it became clear that a TPE program for patients in the context of rare, orphan and chronic disease is also a training program for the caregivers themselves. The design of a collective tool covering all areas of the patients' experience and, therefore, all the necessary multidisciplinary medical and paramedical management, has led care providers to “educate” themselves in the techniques and expectations of their colleagues and to better understand the disease in all its dimensions. Clearer, shared explanations, better mastered by different caregivers, allow them to avoid the collection of inaccurate information, which is a source of confusion and anxiety for families. This common language contributes to build a climate of trust among the different actors and preserves the privileged exchanges they establish. The benefits to caregivers were also important in terms of training, motivation and interaction. Evaluation tools for this dimension of TPE are being developed within the framework of our reinforcement program, which will combine individual and group sessions.

## Conclusion

In conclusion, collective therapeutic educational programs seem to be suitable for ichthyosis. These collective programs should be coupled with individual specific programs taking into account personal needs of patients according to their age. More generally, the contributions of a therapeutic education program in the management of rare genodermatoses may be the following: (1) the trust between the patients, their families and the caregivers was strengthened; (2) the context of the program encouraged self expression, answered questions, and provided mutual aid; and (3) the more self-sufficient families could better manage emergencies. Also, the mobilization strengthened the links between team members, which has improved their training.

## Abbreviations

MAGEC: Maladies Génétiques à Expression Cutanée; MDPH: Maison Départementale des Personnes Handicapées (Departmental Home for Disabled Persons); PAI: Projet d’accueil individualisé (Individualized Integration Plan); TPE: Therapeutic Patient Education; IFAP: Ichthyosis Follicularis Atrichia Photophobia.

## Competing interests

The authors have no conflict of interest to declare.

## Authors’ contributions

HD was involved in establishing conception and design of study, acquisition and interpretation of data, helped to draft the manuscript and participated in revising it critically. SHR was involved in acquisition of data and was involved in writing and revising the manuscript for important intellectual content. CT was involved statistical analysis and revising the manuscript. CB contributed in establishing conception and design of study, and was involved in writing and revising the manuscript for important intellectual content. All authors read and approved the final manuscript.
